# Managing the Adverse Events Associated with Pembrolizumab and Lenvatinib Therapy in Endometrial Cancer

**DOI:** 10.18295/squmj.12.2023.088

**Published:** 2024-05-27

**Authors:** Aref Zribi, Khulood Al Riyami, Hajar S. Al Zahibi, Ikram A. Burney

**Affiliations:** 1 Women Health Program, Sultan Qaboos Comprehensive Cancer Care and Research Centre, University Medical City, Muscat, Oman; 2 Department of Nuclear Medicine, Sultan Qaboos Comprehensive Cancer Care and Research Centre, University Medical City, Muscat, Oman

**Keywords:** Cancer, Endometrial Cancer, Lenvatinib, Pembrolizumab, Adverse Drug Events, Hand-Foot Syndrome, Hypothyroidism, Oman

## Abstract

Endometrial cancer (EC) is the most common gynaecological cancer. The combination of lenvatinib and pembrolizumab has exhibited efficacy as the second line treatment for advanced EC, with a significant benefit in terms of progression free survival (PFS) and overall survival, but the adverse events (AE) profile is complex. AEs associated with the treatment may represent a limitation to this combination. Here, we report the case of a 38-year-old female patient diagnosed with stage IV EC elsewhere, whose disease progressed after the first line of treatment and was referred to a specialised cacncer centre in Muscat, Oman, in 2021. We treated her with the combination of lenvatinib and pembrolizumab. During the course of the treatment, she developed hand-foot syndrome grade III and hypothyroidism grade II. The AEs were managed with supportive medications, dose interruptions, dose reductions and multidisciplinary care, which allowed the continuation of the treatment. The patient achieved a good partial response and an ongoing PFS of more than 12 months.

Endometrial cancer (EC) is the most common gynaecological neoplasm.^1^ Approximately 10–15% of patients with endometrial cancer present with stage IV disease.^2^ The standard of care for first line treatment for women with advanced, recurrent and metastatic endometrial carcinoma is a combination of platinum with paclitaxel.^3^ Up until recently, there was no standard of care treatment for relapse or progression after first line chemotherapy. Lately, a combination of lenvatinib (a tyrosine kinase inhibitor [TKI]) and pembrolizumab (immune checkpoint inhibitor) was approved for second line treatment.^4^ The combination has been approved for use both in mismatch repair deficient and mismatch repair proficient (pMMR) EC.^5^ Although the combination is free of cytotoxic chemotherapy, and hence arguably free of side effects of chemotherapy, a different toxicity profile is observed with the combination treatment.^6^ We present the case of a patient with pMMR EC treated with a combination of pembrolizumab and lenvatinib who developed grade II and III adverse events (AEs) of different kinds. We highlight the importance of management of toxicities while continuing to treat the patient, as there is no efficient third line management.

## Case report

**Figure 1:**
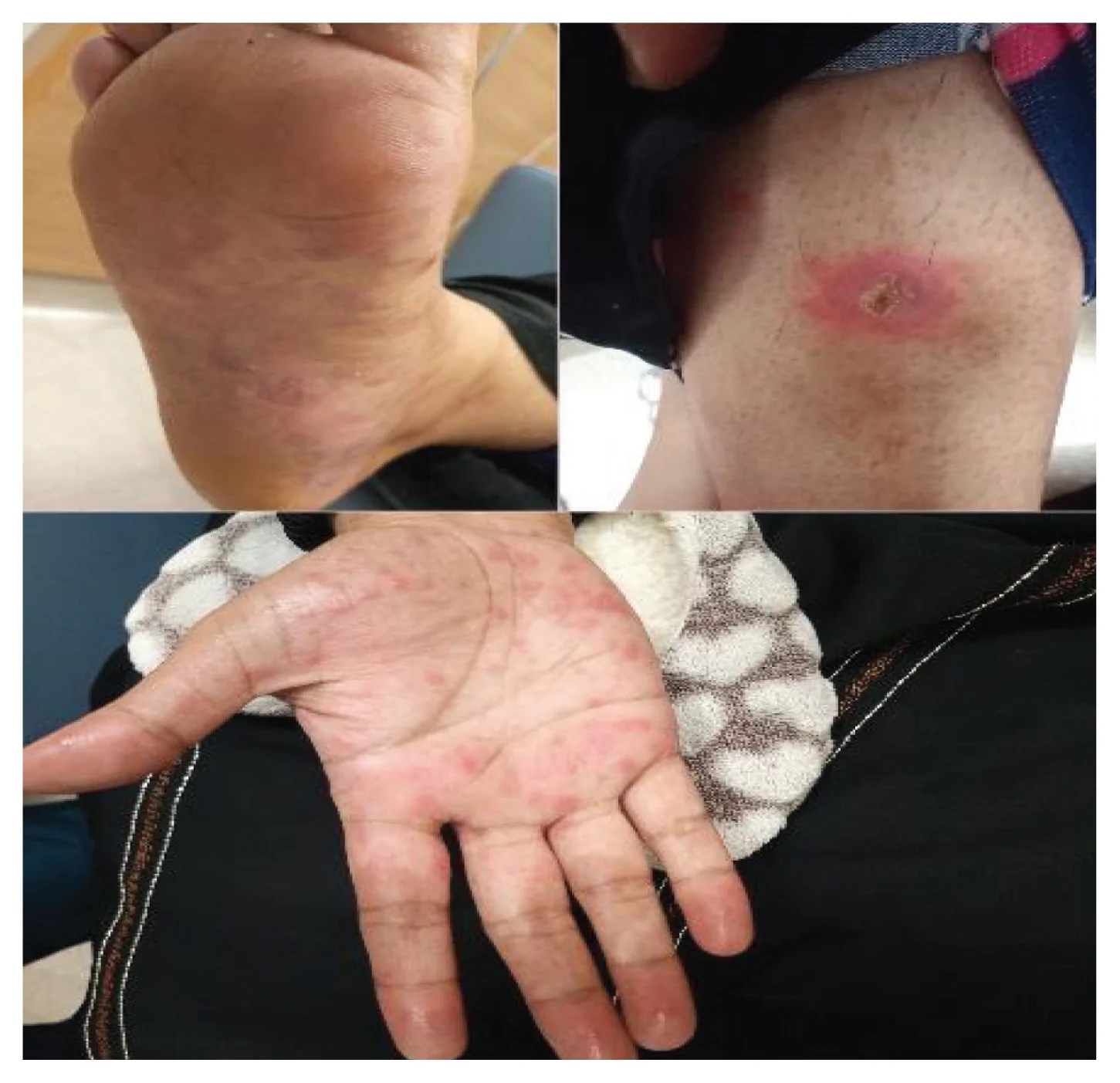
Photographs of hand and foot syndrome developed 3 weeks after starting the lenvatinib plus pembrolizumab treatment.

**Figure 2:**
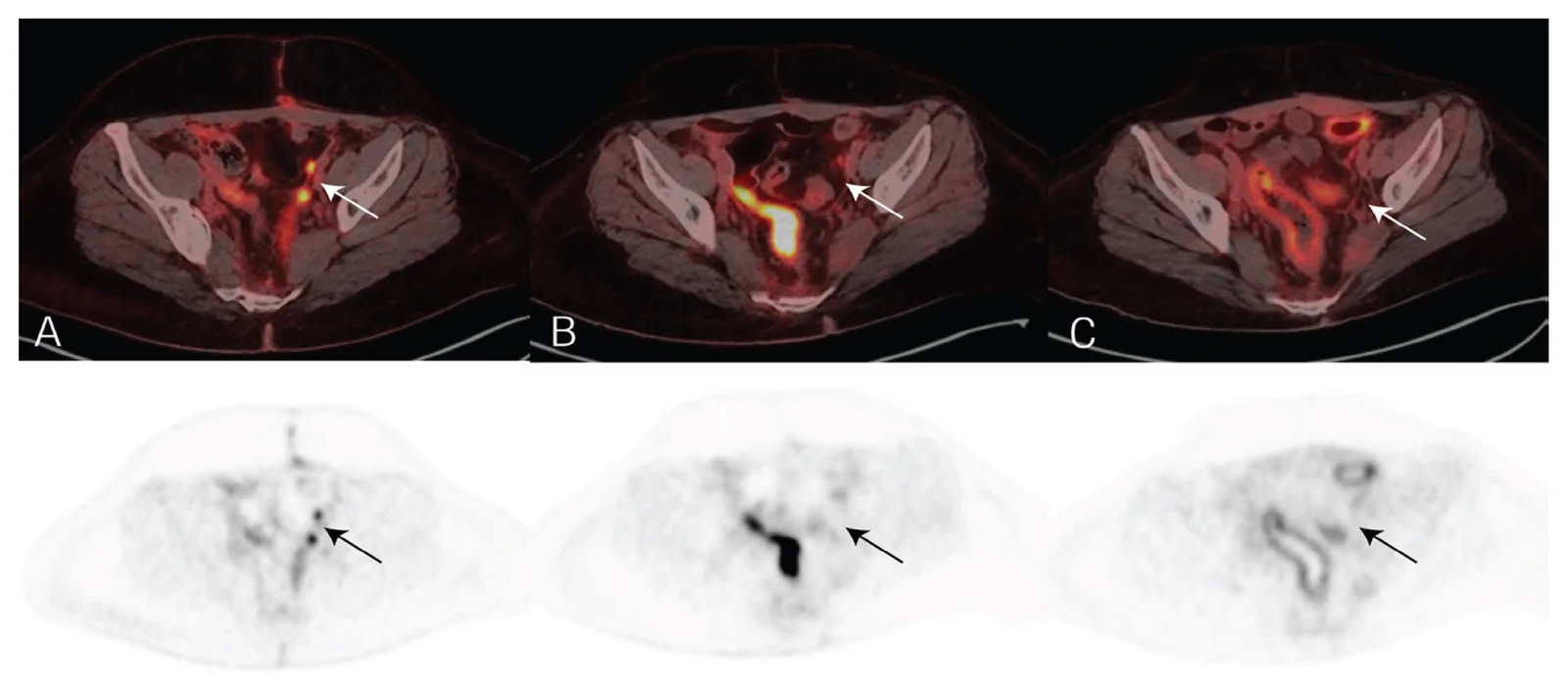
Endometrial carcinoma [18F] FDG-PET/CT at **(A)** base line, **(B)** post-6 cycles immune checkpoint inhibitor (ICI) and **(C)** post-12 cycles of ICI demonstrating avid peritoneal deposits in the left pelvic side wall with interval reduction in metabolic activity in the follow-up studies (arrows); in keeping with partial metabolic response.

**Figure 3:**
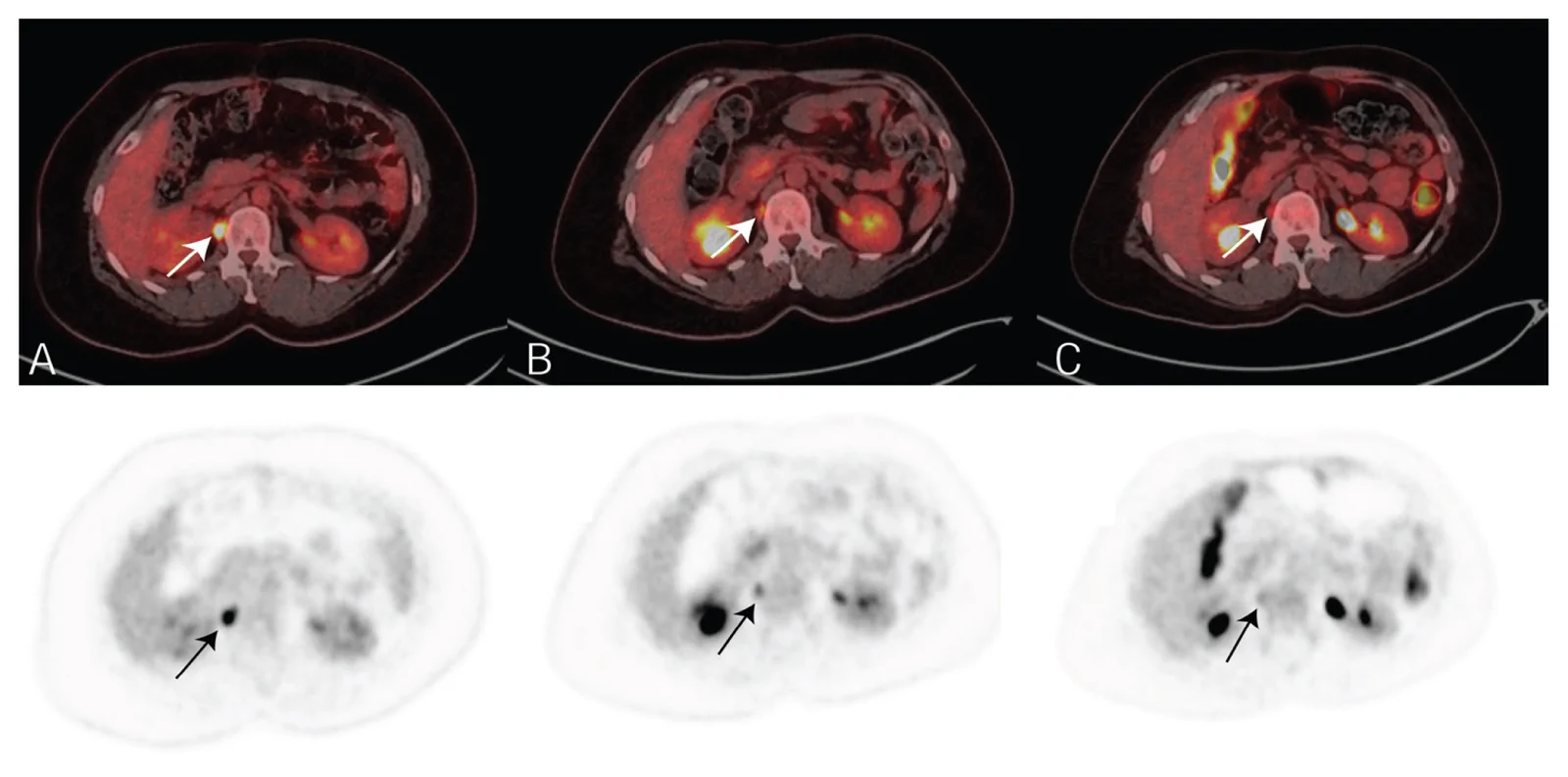
Endometrial carcinoma [18F] FDG-PET/CT at **(A)** base line, **(B)** post-6 cycles immune checkpoint inhibitor (ICI) and **(C)** post-12 cycles of ICI respectively demonstrating an avid right retrocrural lymph node with interval reduction in metabolic activity in the follow-up studies (arrows); in keeping with partial metabolic response.

A 38-year-old female patient was referred to a specialised cancer centre in Muscat, Oman, for further treatment of stage IV EC in 2021. Previously, she had been treated abroad in a tertiary care centre, where she had presented with vaginal bleeding for 3 months. Magnetic resonance imaging (MRI) of the pelvis showed an endometrial mass. Hysteroscopy with biopsy revealed endometrial carcinoma, endometroid type (type I) grade III, the mismatch repair (MMR) protein were all intact on immunohistochemistry (pMMR). A positron emission tomography-computed tomography (PET/CT) scan revealed metabolic uptake in the omentum and peritoneum. The patient was staged to have stage IV EC. The patient received 4 cycles of chemotherapy with a very good partial response and underwent debulking surgery (total hysterectomy, bilateral salpingo-oophorectomy, bilateral pelvic lymph nodes and paraaortic lymph nodes dissection with right diaphragmatic and right lower abdominal wall dissection, bilateral pelvic peritoneum and bladder peritonectomy) with gross residual disease, followed by hyperthermic intra-peritoneal chemotherapy (HIPEC). Although HIPEC is not the standard of care for EC, the patient had received all this treatment before being referred to the current centre. The histopathology report revealed residual viable EC in omental deposits, recto vesical region, and lymph nodes. At this stage, the patient was referred to the current specialised cancer centre in Muscat, Oman, where she received another 6 cycles of chemotherapy, until stable remission and was then commenced on follow-up. Six months later, the disease progressed with multiple FDG avid lesions in the abdomen and pelvis, peritoneal deposits and along the surface of the spleen and the right side of the diaphragm. The patient was commenced on a combination of lenvatinib 10 mg daily (reduced dose) and pembrolizumab 200 mg once every 3 weeks. Three weeks after starting treatment, the patient developed a grade III hand-foot syndrome (HFS) with blisters, skin irritation, pain and the patient was unable to walk [Figure 1]. Lenvatinib was put on hold and the patient was seen by the podiatrist who advised to keep the area dry and clean, daily dressings, avoiding close tight shoes/socks and to use cold therapy to relieve pain. The patient was also treated with urea cream, doxycycline, vitamin B6, ibuprofen and loratadine. Three weeks after withholding the treatment, the HFS improved to grade I and lenvatinib was resumed at the same dose. Seven weeks after starting the treatment, the patient reported fatigue, constipation and weight gain and was found to have hypothyroidism grade II with fatigue, constipation and weight gain (thyroid stimulating hormone = 80 mIU/L, FT4 = 2.9 pmol/L) and the thyroid scan [99mTcO4-] showed a lack of uptake throughout the gland, suggestive of diffuse thyroiditis. Pembrolizumab and lenvatinib were put on hold again and the patient was started on levothyroxine. Subsequently, as the patients achieved euthyroid levels, both drugs were started again after 3 weeks. The dose of lenvatinib was subsequently increased to 14 mg, then to 18 mg and finally to 20 mg every day. The patient achieved a good metabolic response after 6 and 12 cycles of pembrolizumab and continues to be in metabolic response and maintaining quality of life [Figures 2 and 3]. Oral and written consent were taken from the patient for publication purposes.

## Discussion

We report the successful management of AEs associated with a combination of pembrolizumab and lenvatinib in the treatment of a women diagnosed to have stage IV EC, pMMR. The AEs were managed with supportive measures, medications and minimal dose interruptions. The KEYNOTE 775 study reported the comparison of a combination of lenvatinib and pembrolizumab with chemotherapy of treating physician’s choice and showed a statistically significantly clinical benefit in terms of both PFS and OS.^4^ However, the treatment was not free of AEs grade III or higher AEs occurred in 88.9% of the patients; the more frequent being hypertension (37.9%), hypothyroidism (1.2%), diarrhoea (7.6%) and decrease in appetite (7.9%). All patients presented with at least one AE related to the treatment; 66.5% of patients needed a dose reduction after AEs, 33% discontinued the treatment and 69.2% had a temporary interruption to manage toxicities.

Selected AEs of the combination were chosen for detailed post-hoc analyses.^6^ The median time to the starting of most frequent AEs was approximately 3 months after treatment initiation. The median time to first onset of hypertension was 2.1 weeks, diarrhoea was 4.8 weeks and hypothyroidism was 6.1 weeks, the overall incidence of hypertension was 64%, diarrhoea 54% decrease of appetite 44.8%, hypothyroidism 57.4% and HFS was 26%.

The combination of lenvatinib and pembrolizumab has been used to treat other solid tumours such as melanoma, renal cell carcinoma or urothelial cancer.^7^ Vogelzang *et al*. analysed patients with metastatic urothelial carcinoma treated with lenvatinib and pembrolizumab.^8^ Most patients (90%) experienced treatment-related AEs of any grade and 50% had a grade III or IV AEs. Almost 75% of patients had an AE that led to a drug dose adjustment. Dierks *et al*. analysed 8 patients with metastatic thyroid cancer who received a combination therapy of lenvatinib and pembrolizumab.^9^ The most common AEs were hypertension 63%, fatigue 25% and HFS 13%. Grade III/IV toxicities developed in more than half the patients, requiring dose reduction or discontinuation of lenvatinib. In the phase 3 CLEAR trial, patients with advanced renal cell carcinoma were treated with the lenvatinib plus pembrolizumab regimen.^10^ AEs most often occurred within the first 5 months of treatment. The most common AEs of any grade were diarrhoea in 61.4%, hypertension in 55.4%, hypothyroidism in 47.2 %, decreased appetite in 40.3%, fatigue in 40.1% and stomatitis in 34.7%. The most common grade III or higher AEs were hypertension in 27.6%, diarrhoea in 9.7%, weight decrease in 8.0%, proteinuria in 7.7% and fatigue in 4.3%. The analysis of health-related quality-of-life data established that lenvatinib plus pembrolizumab had matching scores compared with those obtained by Sunitinib, especially regarding the time to the definitive deterioration.^11^

Understanding which drug caused AEs helps to determine the subsequent management. The toxicity profile of TKIs and immune checkpoint inhibitor may be different, but there are considerable overlaps, such as skin toxicity, hypothyroidism, transaminitis/hepatitis, etc. It is important to have the knowledge of blood pressure, urine protein levels, thyroid and liver function prior to treatment. The first thing to do when faced with AEs is to grade the toxicity. AEs are reported according to the National Cancer Institute Common Terminology Criteria for Adverse Events (from grade I: mild AEs to IV: life threatening AEs).^12^ If the offending agent is suspected to be lenvatinib and the toxicity is persistent or insupportable grade II or any grade III severity, the recommendation is to stop lenvatinib until resolution to grade ≤I severity AE level. Subsequently, the dose of lenvatinib can be reduced progressively to 14 mg, 10 mg and 8 mg. Alternatively, lenvatinib could be restarted at a lower dose level, for example 10 mg, and the dose could be progressively increased, as was the case with the current patient. Permanent discontinuation of lenvatinib is recommended for any grade IV severity AEs.^13^

Proactive management and close monitoring early after starting the treatment may help conserve patients on combination therapy and enhance outcomes. General management strategies for AEs include supportive medications for symptom management, patient education, dose modification, communication with the work team and the involvement of the relevant specialities.

The median time to first onset of hypothyroidism is 6.1 weeks. Most incidences of hypothyroidism are grade II. Management the strategies for hypothyroidism include concomitant thyroid hormone replacement therapy. Pembrolizumab is allowed to be continued while thyroid replacement therapy is instituted.^6^

In the current patient, hypothyroidism was grade II and the time to onset was 7 weeks after starting pembrolizumab. An endocrinologist was consulted and hypothyroidism was successfully managed with hormone therapy and monitoring thyroid function with every cycle.

Whether hypothyroidism was due to pembrolizumab or lenvatinib is a matter of conjecture. Management depends on clinical judgement. The lenvatinib plus pembrolizumab combination led to a higher frequency (51%) of hypothyroidism than with either monotherapy (8% with pembrolizumab monotherapy in several indications and 22% in patients with unresectable hepatocellular carcinoma treated with lenvatinib monotherapy).^6^

The incidence of hypothyroidism with lenvatinib, when used as a monotherapy is approximately 22%; however, when administered together with pembrolizumab, the incidence increases to 47–57%, which suggests pembrolizumab added toxicity [Table 1]. In the current case, hypothyroidism was most likely a toxicity from pembrolizumab rather than lenvatinib, suggested by diffuse thyroiditis on the scan.

The median time to first onset of HFS is 8 weeks.^6^ Overall incidence was 26% (grade I was 12%; grade II was 11%; and grade III was 3%). Overall, 11.7% of patients were administered at least one medication for HFS. Only 5% of patients had a lenvatinib interruption and 13% of patients had a lenvatinib dose reduction because of HFS. In the current case, HFS was grade III, time of onset was 3 weeks after starting lenvatinib, HFS was managed by withholding lenvatinib, administration of concomitant medication and advice was done by the podiatrist. The HFS improved within 3 weeks. Lenvatinib was re-introduced at a dose of 10 mg and increased to 14 mg, then 18 mg and finally to then 20 mg with a very good tolerance. The appearance of HFS may be correlated with prognosis for numerous TKIs.^14^^,^^15^ Iwasaki *et al*. showed in patients with thyroid cancer treated with lenvatinib that the 24-month overall survival rate was 73.2% in patients with HFS and 52.1% in patients without HFS.^16^ The link between HFS and prognosis of EC has not been reported.

## Conclusion

Lenvatinib plus pembrolizumab combination therapy produces durable and clinically significant activity in advanced and recurrent EC. Close monitoring of patients for AEs is important. The clinical team (physicians, nurses and therapists) should learn how to manage these AEs. Patients should be educated and made aware of the importance of adherence to treatment to optimise its effectiveness, about the mechanism of action, common toxicities and strategy of management. This case should provide an illustration of good management of AEs for patients receiving lenvatinib plus pembrolizumab combination therapy.

## Figures and Tables

**Table 1:** Most frequent adverse events in the different studies with lenvatinib and pembrolizumab^4^^,^^6^^,^^17^^,^^18^,^19^

	Lenvatinib in TC17	Lenvatinib in HCC18	Pembrolizumab + lenvatinib in RCC10	Pembrolizumab + lenvatinib in EC4,6
HFS	31.8%	29.4%	28.7%	26%
Hypothyroidism	-	22.5%	47.2%	57.4%
Diarrhea	59.4%	24.6%	61.4%	54.2%
Hypertension	67.8%	36.8%	55.4%	64%
Fatigue	59.4%	34.5%	40%	33%

AEs = adverse events; TC = thyroid cancer; HCC = hepatocellular carcinoma; RCC = renal cell carcinoma; EC = endometrial carcinoma; HFS: Handfoot syndrome.
